# Hypokalemia induced myopathy as first manifestation of primary hyperaldosteronism - an elderly patient with unilateral adrenal hyperplasia: a case report

**DOI:** 10.4076/1757-1626-2-6813

**Published:** 2009-07-16

**Authors:** Panagiotis Kotsaftis, Christos Savopoulos, Dimitrios Agapakis, George Ntaios, Valentini Tzioufa, Vasilios Papadopoulos, Epaminondas Fahantidis, Apostolos Hatzitolios

**Affiliations:** 1 First Propedeutic Department of Internal Medicine, AHEPA Hospital, Aristotle UniversityS. Kiriakidi1, Thessaloniki, 54636Greece; 2 Department of Pathology, AHEPA Hospital, Aristotle UniversityS. Kiriakidi1, Thessaloniki, 54636Greece; 3 First Propedeutic Surgical Clinic, AHEPA Hospital, Aristotle UniversityS. Kiriakidi1, Thessaloniki, 54636Greece

## Abstract

**Introduction:**

Primary hyperaldosteronism is only rarely caused by unilateral adrenal hyperplasia.

**Case presentation:**

A 73-year-old hypertensive Greek man (on 10 mg amlodipine for the last ten years) presented in the emergency department with severe muscle weakness of all limbs. The initial physical and laboratory examination revealed normal blood pressure, muscle weakness, severe hypokalemia, sinus rhythm and U wave, rhabdomyolysis and metabolic alkalosis. The patient was immediately treated with intravenous administration of potassium-rich solutions, 25 mg spironolactone with progressive dose titration up to 100 mg. Because of high arterial blood pressure, irbesartan was added. On day 6, muscle weakness was completely restored with decrease of arterial blood pressure and further improvement of laboratory tests. The combination of hypokalemia with arterial hypertension raised the suspicion of primary hyperaldosteronism; therefore, we performed abdomen computed tomography scan, which revealed a nodular mass (15 mm in diameter) in the left adrenal gland. Plasma renin activity was in the lower normal range with a three-fold increase of plasma aldosterone concentration. We performed total resection of the left adrenal gland and the histopathological examination revealed hyperplasia of the left adrenal gland.

**Conclusion:**

This report presents a rare case of an elderly patient under antihypertensive treatment the last ten years for essential hypertension, who admitted to our emergency department with hypokalemia - induced myopathy as first manifestation of primary hyperaldosteronism due to unilateral adrenal hyperplasia.

## Introduction

Primary hyperaldosteronism (PH) or Conn’s disease was first described by Jerome W. Conn in 1955 in a 34-year-old woman with arterial hypertension, intermittent paralysis, hypokalemia and metabolic alkalosis [1-3]. The most frequent findings of PH are arterial hypertension, hypokalaemia, suppressed plasma renin activity (PRA) and increased plasma aldosterone concentration (PAC) [1-3]. In most cases (>90%), PH is attributed to bilateral adrenal hyperplasia (idiopathic hyperaldosteronism-IHA) or aldosterone-producing adenoma (APA). Unilateral adrenal hyperplasia (UAH) is a rare cause of PH [1,2].

In this paper we report the case of a 73-year-old man with a history of arterial hypertension for the last 10 years treated with 10 mg amlodipine. He presented to our emergency department with severe muscle weakness of all limbs, which was actually the first manifestation of PH caused by UAH.

## Case presentation

A 73-year-old hypertensive Greek man (on 10 mg amlodipine for the last ten years) presented to our emergency department with severe muscle weakness of all limbs. The initial physical and laboratory examination revealed arterial blood pressure (140/80 mmHg), muscle weakness, severe hypokalemia (K^+^ = 1.6 meq/l), sinus rhythm and U wave, rhabdomyolysis (Creatinine Phosphokinase, CPK = 7463 U/l) and metabolic alkalosis (arterial blood gasses: pH = 7,546, pCO_2_ = 43, pO_2_ = 61, HCO_3_^-^ = 37, SO_2_ = 93%). Because of the low serum potassium level and clinical suspicion of PH and to the fact that all the necessary laboratory examinations had been already done, the patient was immediately treated with intravenous administration of potassium-rich solutions, 25 mg spironolactone with progressive dose titration up to 100 mg.

On day 3 and under a daily dose of 100 mgs spirinolactone, the laboratory findings were: K^+^ = 3 meq/l, CPK = 3604 U/l, arterial blood gas: pH = 7.571, pCO_2_ = 37.2, pO_2_ = 70.1, HCO_3_^-^ = 34.5, SO_2_ = 97.3%. Because of uncontrolled hypertension (185/105 mmHg), 300 mgs irbesartan were further added. On day 6, muscle weakness was completely restored with decrease of arterial blood pressure (155/90 mmHg) and further improvement of laboratory tests (K^+^ = 3.9 meq/l, CPK = 1362 U/l, arterial blood gasses: pH = 7.49, pCO_2_ = 38.6, pO_2_ = 77.5, HCO_3_^-^ = 29.1, SO_2_ = 97.6%).

**Figure 1.**
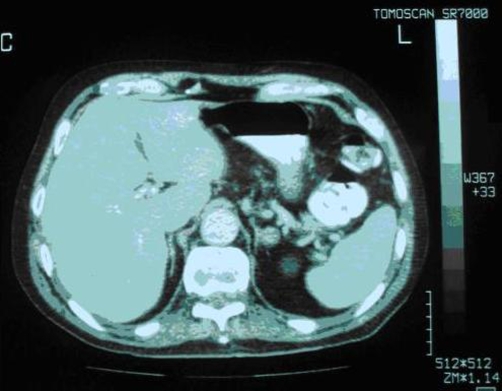
Nodular mass (diameter approximately 15 mm) on the left adrenal gland. The right adrenal gland appears normal.

The combination of hypokalemia with arterial hypertension raised the suspicion of PH; therefore, we performed an abdominal Computed Tomography scan (CT), which revealed a nodular mass (15 mm in diameter) in the left adrenal gland (Figure 1). PRA was in the lower normal range (0.7 ng/ml/h, normal values: 0.2-2.8 ng/ml/h) with a three-fold increase of PAC (498 pg/ml, normal values: 10-160 pg/ml) and the ratio PAC/PRA was 71.14 ng/dl/ng/ml/h. Moreover plasma concentrations of adrenocorticotropic hormone (ACTH) and cortisol were (34 pg/ml, normal values: 0-40 pg/ml) and (348 nmol/l, normal values: 171-536 nmol/l) respectively. Our suspicion of PH was further supported by the combination of hypokalemia and hyperkaliuria (64 meq/24 hrs, normal values: 23-127 meq/24 hrs), whereas urinary sodium concentration was within normal range (106 meq/24 hrs, normal values: 40-220 meq/24 hrs). We notice that all the above assays, except the urine analysis done prior to starting any treatment. On account of the clinical suspicion of unilateral APA of the left adrenal gland we did not supply the salt loading suppression tests (acute into 4 hours, intravenous saline loading as well as a 4-day fludrocortisone administration), in order to confirm or exclude the diagnosis of PH. Furthermore, we did not perform specific imaging examinations including Magnetic Resonance Imaging (MRI) of the adrenal glands and scintigraphy with iodine-131.

**Figure 2.**
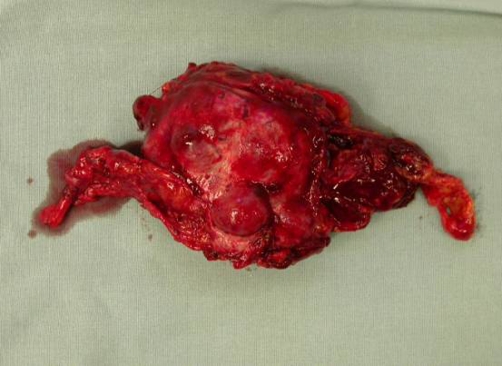
Operative specimen of the left adrenal gland.

**Figure 3.**
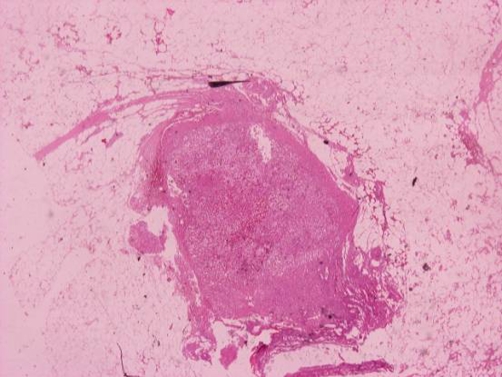
An adrenal cortical nodule in adipose tissue (HE × 40).

**Figure 4.**
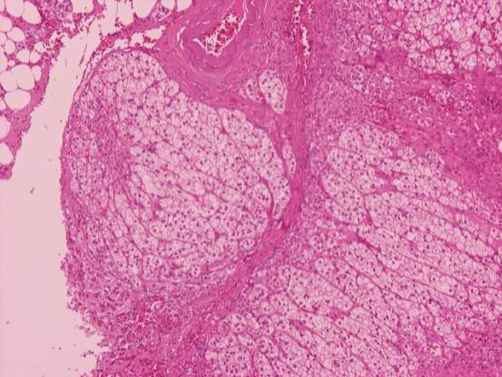
Fine connective tissue septa separate adrenal cortical clear cells nodules (HE × 100).

We performed total resection of the left adrenal gland (Figure 2). The histological findings of the specimen were consistent with mild grade nodular adrenal cortical hyperplasia and confirmed the diagnosis of PH. The microscopy revealed many adrenal tissue specimens with cortical hyperplasia. Nodular pattern and nodules were separated by fine connective tissue septa. Most cells were clear cells and form cords and nests. In one specimen an adrenal cortical nodule in adipose tissue was observed. Focally in connective tissue bands perivascular infiltration was observed (Figures 3, 4).

Six months after discharge, the patient is asymptomatic, normotensive and normokalemic without any treatment.

## Discussion

PH constitutes the most frequent cause of secondary arterial hypertension [1,2]. The incidence of PH ranged between 0.05-2% in the past decades but currently ranges between 5-10%, probably due to more efficient diagnostic approach nowadays [1,2,4,5]. The rate of PH among patients with resistant hypertension is approximately 20% [5] and the age of diagnosis is usually between 20-60 years [2]. Furthermore, a recent study reported that all patients with PH due to UAH were hypertensive and the median age was 49 (range: 10-62) years [6]. Table 1 presents the frequency of the different causes of PH [1,2].

**Table 1.** Causes of primary hyperaldosteronism

Causes of primary hyperaldosteronism	Frequency (%)
Idiopathic hyperaldosteronism	65
Aldosterone-producing adenoma	30
Primary unilateral adrenal hyperplasia	3
Aldosterone-producing adrenocortical carcinoma	<1
Aldosterone-producing ovarian tumor	<1
Familiar hyperaldosteronism	<1

Severe muscle weakness is a classic manifestation of PH and is usually related to coexistent hypokalemia [7]. On the contrary, in a review study of 30 patients with PH due to UAH, myopathy is not reported as a characteristic manifestation [6]. In the majority of cases presenting with myopathy, paralysis commonly affects the limb muscles [7]. Our patient presented with severe muscle weakness of all limbs and plasma potassium levels was 1.6 meq/l. In a study by Huang et al on patients with PH, plasma potassium was lower in patients with paralytic myopathy compared to those without (1.8 ± 0.3 mmol/L vs. 2.3 ± 0.4 mmol/L respectively) [7]. Furthermore a study by Crawhall et al. concluded that patients with severe hypokalemia - such as observed in PH accompanied with muscle weakness - may have elevated CPK directly related to preexisting hypokalemia [8]. Table 2 shows the change of potassium and CPK levels during the first week of hospitalization of our patient.

**Table 2.** Variation of serum potassium and CPK at the 1^st^ week of hospitalization

Variation of serum potassium (meq/lt) and CPK (mg/dl)							
Days	1	2	3	4	5	6	7
Potassium	1,6	2,2	3	3,2	3,5	3,9	4,4
CPK	7463	6531	3604	3007	2816	1362	575

Hypokalemia is one of the most frequent findings of PH [2]. In the study by Goh et al, most patients with PH due to UAH were hypokalemic at presentation, with a median serum potassium level of 2.8 (range: 1.4-3.9) mmol/l, whereas in comparison with our patient, only one patient had potassium levels lower than 1.6 mmol/l and two equal to 1,6 mmol/l [6]. In 1965, Conn et al. first suggested that hypokalemia is not a prerequisite for the diagnosis of PH something that was later confirmed by other studies, which showed that most patients with PH were normokalemic [2]. Recently another Greek study showed that hypokalemia was present in 83 (45.6%) patients with resistant hypertension due to PH [5]. On the contrary, in a study by Pekarske et al, [4] hypokalemia constituted one of the most frequent findings among patients with PH. Moreover, Loh et al. reported that among hypertensive patients with primary hyperaldosteronism, hypokalemia can present many years after the appearance of arterial hypertension [9].

There are some specific tests, which confirm the diagnosis of PH [1,2,10]. In our case we did not perform any of these tests, on the one hand because of the severity of hypokalemia and the clinical status of the patient and on the other because of the obvious representation of a nodular mass on the left adrenal gland in abdominal CT. On the contrary, we administered at once from the 1^st^ day of hospitalization, spironolactone (25 mg per day) with progressive titration of dose in the next days. Furthermore, it is known that for the reliability of these specific tests, it is necessary the interruption of spironolactone at least for six weeks, or its administration when these tests have completed [1,2]. In addition, in a study by Karagiannis et al, [11] was reported that in many cases like ours, when the clinical status of the patient suffered PH is very severe, an urgent therapeutic procedure is necessary.

Finally, in accordance with other findings, in most cases, the imaging control (CT-MRI or scintigraphy) contributes to the diagnosis and helps in deciding the appropriate mode of treatment [1]. In the study by Young et al, [12] was reported that a unilateral adrenal macroadenoma (>1 cm) detected by CT is highly suggestive of APA, so that further evaluation to distinguish between APA and IHA is probably not necessary. On the contrary, in the study by Tamura et al, [13] it was reported that in many cases of small size APA (microadenomas) are undetectable by classical imaging tests for a long period and it demands further investigation with specific tests. In accordance to the laboratory and imaging examinations (nodular mass diameter approximately 15 mm on the left adrenal gland) and personal history of the patient (arterial hypertension since last 10 years), we did not perform specific tests. The patient was transferred to the surgical department and had unilateral adrenal surgery. The histopathological examination revealed hyperplasia of the left adrenal glad and confirmed the diagnosis of PH.

PH is only rarely caused by UAH [3]. The possibility that UAH can cause PH was suggested by Ross in 1965 [6]. Omura et al. reported that the prevalence of APA, IHA and UAH was 4.9%, 1.2% and 0.1% respectively [14]. UAH constitutes a rare cause of PH, which is difficult to diagnose because imaging results are unreliable [15]. This rare cause of PH usually mimicks an adrenal adenoma and is difficult to diagnose prior to resection and histopathological examination [3]. We notice, that in our case although the CT showed a nodular mass on the left adrenal gland, diameter approximately 15 mm, eventually the histopathological examination revealed hyperplasia of the left adrenal glad. The pathogenesis of UAH is not clearly understood. This disorder is thought to be a precursor of adenoma formation or an intermediate state between APA and IHA [15].

The treatment of choice for UAH is unilateral total adrenalectomy [15,16] which may be complicated by postoperative hyperkalemia due to secondary hypoaldosteronism; thus, serum potassium levels should be monitored weekly for 4 weeks [1]. Typically, arterial hypertension decreases substantially within 1 to 3 months without antihypertensive treatment [1,2]. Nevertheless, a recent study reported that patients with PH secondary to UAH have excellent outcomes after surgical treatment [6]. Our patient did not develop any complication. Six months after discharge, the patient is under periodical follow up. The levels of plasma potassium as well as the arterial blood pressure are normal without pharmaceutical treatment.

## Conclusion

Summarising, this report presents a rare case of an elderly patient under antihypertensive treatment the last ten years for essential hypertension, who admitted to our emergency department with hypokalemia - induced myopathy as first manifestation of PH due to UAH.

## Abbreviations

ACTH: adrenocorticotropic hormone
APA: aldosterone-producing adenoma
CPK: Creatinine Phosphokinase
CT: Computed Tomography
IHA: idiopathic hyperaldosteronism
MRI: Magnetic Resonance Imaging
PAC: plasma aldosterone concentration
PH: Primary hyperaldosteronism
PRA: plasma renin activity
UAH: Unilateral adrenal hyperplasia
